# Type 2 diabetes mellitus is associated with increased risks of sarcopenia and pre-sarcopenia in Chinese elderly

**DOI:** 10.1038/srep38937

**Published:** 2016-12-13

**Authors:** Taotao Wang, Xiao Feng, Jingjing Zhou, Hongyan Gong, Song Xia, Qing Wei, Xu Hu, Ran Tao, Lei Li, Frank Qian, Li Yu

**Affiliations:** 1 Department of Endocrinology and Clinical Nutrition, The Affiliated Hospital of Jiangsu University, Zhenjiang, China; 2 Jiangsu University Library, Zhenjiang, China; 3 Pritzker School of Medicine, University of Chicago, USA

## Abstract

Sarcopenia is a condition characterized by progressive and generalized loss of skeletal muscle mass and function. In this study, we used a cross-sectional study with 1090 community-dwelling Chinese citizens aged 60 years and older to evaluate the association of type 2 diabetes mellitus (T2DM) with the risk of sarcopenia and pre-sarcopenia. Sarcopenia was defined using the Asian Working Group for Sarcopenia (AWGS) criteria that include both muscle mass and muscle function/physical activity. Pre-sarcopenia was defined as having low skeletal muscle index but with normal muscle/physical activity. The prevalence of sarcopenia and pre-sarcopenia was significantly higher in T2DM patients than in healthy controls (14.8% vs. 11.2%, p = 0.035 for sarcopenia, and 14.4% vs. 8.4%, p = 0.002 for pre-sarcopenia). In multivariate logistic regression analyses adjusting by age, gender, anti-diabetic medication, energy intake, protein intake, physical activity, and visceral fat area, we found that Chinese elderly with T2DM exhibited significantly increased risks of sarcopenia (OR = 1.37, 95% CI = 1.02–2.03) and pre-sarcopenia (OR = 1.73, 95% CI = 1.10–2.83) compared to non-diabetic individuals. This is the first study to evaluate the association of T2DM with the risks of sarcopenia and pre-sarcopenia in China. Among a group of community-dwelling Chinese elderly, T2DM was significantly associated with increased risks of sarcopenia and pre-sarcopenia.

Sarcopenia is a condition characterized by progressive and generalized loss of skeletal muscle mass and function^1^,^2^. Sarcopenia is primarily a disease of the elderly and is associated with increased risks of falls and fractures, mobility disorders, disabilities, complications, infection, metabolic disorders, poor quality of life, and mortality^3^,^4^,^5^,^6^. The prevalence of sarcopenia as reported in literature varies considerably, ranging from 1–29% in community-dwelling populations and 14–33% in long-term care populations^7^,^8^,^9^. This discrepancy was mostly due to varied definitions of sarcopenia and different races/ethnicities. Sarcopenia was originally defined as a decrease in muscle mass related to aging^10^. However, latter studies have shown that declined muscle strength is more important than reduced muscle mass in predicting morbidity and mortality in the elderly^11^,^12^,^13^,^14^,^15^, which led to the new criteria for diagnosing sarcopenia developed by the European Working Group on Sarcopenia in Older People (EWGSOP) in 2010: decrease of muscle strength, reduced muscle mass, and impaired physical performance^16^. Recently, the Asian Working Group for Sarcopenia (AWGS) adopted the same criteria and proposed a new diagnostic algorithm for sarcopenia in Asians by recommending Asian-specific cutoff values for muscle mass measurements, handgrip strength, and usual gait speed^17^. There have been very few studies that applied the AWGS criteria to estimate the prevalence of sarcopenia in Asians, particularly in Chinese populations^18^,^19^.

China accounts for nearly 20% of the global population. The number of Chinese aged 60 or older topped 200 million in 2013 and is projected to grow to over 300 million in 2025. Sarcopenia and its associated comorbidities are a major health issue for the rapidly aging Chinese population. An accurate estimate of the prevalence of sarcopenia in Chinese population will facilitate intervention in clinical practice to improve health care outcomes of the elderly. An earlier study using the old criteria of muscle mass to define sarcopenia reported a prevalence of 12.3% for Chinese men and 7.6% for Chinese women aged 70 years and older^20^. Two recent studies applied the new AWGS algorithm to define sarcopenia: Gao *et al*.^18^ reported an overall prevalence of 9.8% (12.0% in women and 6.7% in men) in 612 community-dwelling elderly aged 60 years or older and Han *et al*.^19^ reported a prevalence of 6.4% in men and 11.5% in women in 1,069 suburb-dwelling Chinese elderly aged 60 years or older. In our current study, we used the new AWGS criteria to estimate the prevalence of sarcopenia and pre-sarcopenia in an urban-dwelling elderly population stratified by gender.

More importantly, in this study, we also determined the association of Type 2 diabetes mellitus (T2DM) with sarcopenia and pre-sarcopenia. T2DM is an increasingly prevalent disease worldwide. A systematic review and meta-analysis showed that T2DM patients are at increased risks of physical disability, including disability of mobility, activities of daily living and instrumental activities of daily living^21^. Several studies have also shown that T2DM patients exhibited greater loss of muscle mass, strength, and functional capacity with aging than non-T2DM individuals^22^,^23^,^24^,^25^,^26^,^27^. However, to date, no study has investigated the association of T2DM and sarcopenia using the new AWGS criteria in Asians. This current study will fill the gap. We hypothesize that T2DM is associated with an elevated risk of sarcopenia and pre-sarcopenia among Chinese elderly.

## Results

### Participant characteristics

Table 1 shows the comparison of demographic, anthropometric, body composition parameters and diabetes-related indexes between T2DM patients and controls stratified by gender. The patients and controls were well matched on age. The total body fat mass (FM), trunk FM, appendicular fat mass (AFM), and percentage of fat were all significantly higher in patients than controls in men, but not in women. The frequency of overweight people was significantly higher in patients than controls in men (47.4% vs. 32.2%, p = 0.003), and the frequency of overweight and obese women was higher in patients than controls (31.7% vs. 24.4%, p = 0.109 for overweight, and 15.8% vs. 7.1%, p = 0.003 for obesity) in women. Likewise, the rate of visceral obesity was significantly higher in patients than in controls in both men (93.1% vs. 48.0%, p < 0.001) and women (29.2% vs. 19.6%, p < 0.001). There was no significant difference in ASM, SMI, and handgrip strength between patients and controls in men or women. However, the gait speed was significantly lower in patients than in controls in both men (1.08 ± 0.22 vs. 1.23 ± 0.18, p < 0.001) and women (1.07 ± 0.26 vs. 1.26 ± 0.16, p < 0.001).

### Prevalence of sarcopenia and pre-sarcopenia

Table 2 shows the prevalence of sarcopenia and pre-sarcopenia in T2DM patients and controls stratified by gender. The prevalence of sarcopenia in controls was 11.2% (13.1% in men and 9.6% in women) and the difference between genders did not reach statistical significance (p = 0.1). The prevalence of pre-sarcopenia in controls was 8.4% and there was significantly higher rate of pre-sarcopenia in men than women (11.4% vs. 5.8%, p = 0.002). The prevalence of sarcopenia in patients was 14.8% (17.2% in men and 12.5% in women, p = 0.104). The prevalence of pre-sarcopenia in patients was 14.4% overall and the prevalence was significantly higher in men than in women (19.0% vs 10.0% p = 0.03). Compared between patients and controls, the prevalence of both sarcopenia (14.8% vs. 11.2%, p = 0.035) and pre-sarcopenia (14.4% vs. 8.4%, p = 0.002) was significantly higher in T2DM patients than in controls.

We then stratified the study participants into two groups based on age: those aged 60–69 years (n = 578) and those aged 70 years or older (n = 512). Table 3 shows that prevalence of sarcopenia in these two age groups stratified by gender. As expected, the prevalence of sarcopenia was significant greater in older participants (70 years or older) than in younger participants (60–69 years old) in overall participants (17.4% vs. 7.3%, p < 0.001), in men (19.8% vs. 8.3%, p < 0.001), and in women (15.0% vs. 6.4%, p = 0.001).

Finally, we performed multivariate logistic regression analyses to assess the association of T2DM with sarcopenia, pre-sarcopenia, and low SMI (sarcopenia plus pre-sarcopenia) (Table 4). We found that Chinese elderly with T2DM exhibited significantly increased risks of sarcopenia (OR = 1.37, 95% CI = 1.02–2.03), pre-sarcopenia (OR = 1.73, 95% CI = 1.10–2.83), and low SMI (OR = 1.64, 95% CI, 1.25–2.07) compared to non-diabetic individuals after adjusted by age, gender, anti-diabetic medication, energy intake, protein intake, physical activity, and visceral fat area. When stratified by age, the risks of T2DM with low SMI appeared to be slightly stronger in older people. In individuals aged 60–69 years, T2DM patients were at increased risks of sarcopenia (OR = 1.47, 95% CI,0.61–2.75, p = 0.35), pre-sarcopenia (OR = 1.57, 95% 0.74–2.79, p = 0.26), and low SMI (OR = 1.41, 95% CI, 0.87–2.35, p = 0.053), although none of these associations reached statistical significance due to a smaller sample size. In individuals aged 70 years or older, T2DM patients were at a borderline significantly increased risk of sarcopenia (OR = 1.51, 95% CI, 0.72–2.25, p = 0.17) and significantly increased risks of pre-sarcopenia (OR = 2.17, 95% CI, 1.11–4.03, p = 0.001) and low SMI (OR = 2.23, 95% CI, 1.18–2.85, p = 0.000) compared to non-diabetic individuals.

## Discussion

To our knowledge, this is the first study to examine the prevalence of sarcopenia in T2DM patients and assess the association between T2DM and sarcopenia in China according to the AWGS criteria. Our data showed that patients with T2DM had a higher risk of sarcopenia, pre-sarcopenia and low skeletal muscle index than those without diabetes.

We found that the prevalence of sarcopenia was 11.2% in controls (13.1% in men and 9.6% in women), which is in line with and slightly higher than two recent reports in Chinese populations using AWGS criteria. Gao *et al*.^18^ reported an overall prevalence of 9.8% (12.0% in women and 6.7% in men) in community-dwelling rural and urban elderly and Han *et al*.^19^ reported a prevalence of 6.4% in men and 11.5% in women in suburb-dwelling elderly. It should be pointed out that we used a multi-frequency, 8-point tactile electrode bioelectrical impedance analysis (BIA) system to measure whole body composition, which allows more reliable measurement of muscle mass compared to methods used in previous studies. We also found a non-significant higher rate of sarcopenia in men than women, which appeared to be different from the other two studies, although none of these comparisons reached statistical significance. Further larger studies are needed to clarify whether there is gender difference in the prevalence of sarcopenia in Chinese populations.

The major observation of this study is that patients with T2DM had higher rate of sarcopenia than healthy people and T2DM was associated with a 1.56-fold increased risk of sarcopenia. It has long been known that T2DM patients are at increased risks of physical disability in elderly^21^ and a number of studies have also shown that T2DM patients exhibited greater loss of muscle mass, strength, and functional capacity with aging than non-T2DM individuals^22^,^23^,^24^,^25^,^26^,^27^, although no study has specifically determined the association of T2DM with sarcopenia using the new EWGSOP and AWGS criteria. An earlier report from the Korean Health, Aging, and Body Composition Study examined leg and arm muscle mass and strength at baseline and 3 years later in 1,840 older adults aged 70–79 years^22^. The authors found participants with T2DM (n = 305) showed greater declines in the leg muscle mass and strength compared with those without diabetes. Leg muscle quality, expressed as maximal strength per unit of muscle mass, also declined more rapidly in T2DM patients. In contrast, changes in arm muscle strength and quality were not different between those with and without diabetes. These observations were consistent with our observations that T2DM patients exhibited dramatically lower gait speed in both men and women (p < 0.001), but the handgrip strength was similar between those with and without T2DM (Table 1). Another report from the Korean Sarcopenic Obesity Study (KSOS) including 810 subjects (414 patients with T2DM and 396 control subjects), in which sarcopenia was defined using the skeletal muscle index only, found the prevalence of sarcopenia in patients with T2DM more than doubled that in the control group (15.7% vs. 6.9%)^23^. Our data are consistent with these reports, however, both the study above study one or two composition of the sarcopenia between diabetes and non-diabetes without using the new diagnosing criteria of sarcopenia developed by EWGSOP or AWGS. Our study is the first to use the new AWGS criteria (including both muscle mass and muscle strength/physical performance) in Asians.

We also estimated the prevalence of pre-sarcopenia (low SMI only) according to the definition by AWGS. We found that patients with T2DM, particularly those aged 70 years or older, had a 2.3-fold increased risk of pre-sarcopenia compared to non-diabetic individuals. These results illustrate that older diabetic adults under normal body weight and physical performance were also associated with higher risk of loss of skeletal muscle mass compared with non-diabetic counterparts. Moreover, we found that over 90% of study participants were not aware of their pre-sarcopenia status during screening (data not shown). Unawareness to the loss of skeletal muscle mass may delay appropriate preventative treatments and contribute to accelerate the process of skeletal muscle mass attenuation and onset of adverse outcomes such as metabolic disorders, falls and fractures. Thus it is important to determine and inform individuals of their pre-sarcopenia and sarcopenia statuses during screening.

The underlying mechanisms for higher prevalence of sarcopenia and pre-sarcopenia in older people with T2DM are not clear. Decreased IGF-1 level was associated with muscle growth impaired and low muscle mass in healthy people and in patients with T2DM^28^,^29^,^30^,^31^. Therefore, decreased IGF-1 level may be a potential biological mechanism linking link sarcopenia and low muscle mass with T2DM. Additional potential mechanisms linking T2DM and sarcopenia include insulin resistance, inflammatory cytokines, oxidative stress, and mitochondrial dysfunction^28^,^29^,^30^. Further basic and clinical studies are needed to uncover the molecular mechanisms underlying increased sarcopenia risk in T2DM patients.

Our study is the first to report the association between T2DM and increased risks of sarcopenia and pre-sarcopenia in Chinese elderly using the AWGS criteria. Our study gave the most accurate estimate of the prevalence of sarcopenia and pre-sarcopenia in Chinese elderly and T2DM patients. These data can help provide evidence-based recommendation for the prevention efforts to combat the development of sarcopenia and pre-sarcopenia among older individuals with T2DM through better blood glucose control and increased physical activity. There were a few limitations in the present study. Firstly, the sample size of T2DM patients was relatively small. The reason for the limited sample size was because we set strict inclusion criteria. Old-aged T2DM patients had high prevalence of serious diabetes-related complications such as cerebral stroke, end-stage renal disease and cardiac insufficiency, which limited their physical activity and total energy intake. We excluded those patients with these diabetes-related complications to reduce the confounding factors on measuring muscle mass and function. Nevertheless, we have achieved our main goal to estimate the prevalence of sarcopenia and pre-sarcopenia and established the association between T2M and increased risks of sarcopenia and pre-sarcopenia in Chinese elderly. We will continue to expand the sample size of T2DM to study nutritional and physical interventions to sarcopenia and pre-sarcopenia in elderly Chinese adults with T2DM. Secondly, this is a retrospective cross-sectional study that cannot determine the causality between T2DM and increased risk of sarcopenia. There may be comorbidities in T2DM patients that limit their physical activity whereas community-dwelling controls may have healthier diet and exercise habits, and these variables may confound the association of T2DM and sarcopenia. Thirdly, recall bias and selection bias are inherited limitations of retrospective case control studies. Although we matched cases and controls on age and gender, and adjusted for a number of significant variants in multivariate logistic regression, we cannot completely rule out these biases. Only prospective studies can overcome these limitations. Lastly, we used a food frequency questionnaire for the prevention and management of osteoporosis (FFQPOP) developed by Uenishi *et al*.^31^ because it is a simple questionnaire to estimate the intake of calcium and other nutrients, which is suitable for the main purpose of our cohort to assess the muscle and bone health of Chinse elderly. However, the correlation coefficients of energy and protein intake between the FFQPOP and the conventional diet record method are modest. Researchers should be cautious when using the FFQPOP to estimate energy and protein intake in their studies.

In conclusion, this is the first study to show that patients with T2DM in China are associated with higher risks of sarcopenia, pre-sarcopenia and low muscle mass using the new AWGS criteria. Our data highlight the need for prevention efforts to combat the development of sarcopenia among older individuals with T2DM through better blood glucose control and increased physical activity.

## Methods

### Study participants and data collection

A total of 1090 subjects aged 60–95 years were included in this study, among whom were 236 (152 men, 84 women) patients with T2DM and 854 healthy community-dwelling volunteers. All participants were living in Zhenjiang city of Jiangsu province, China. The T2DM patients were admitted to our hospital for glycemic control between December 2014 and November 2015. T2M was defined as having fasting capillary blood glucose level (>6.1 mmol/L) or plasma glucose measurement (FPG > 7.0 mol/L) and/or 2-h postprandial blood glucose >11.1 during an oral glucose tolerance test in accordance with World Health Organization (WHO) 2006 criteria. Some previously diagnosed diabetes patients are currently using oral hypoglycemic medications or insulin. Metabolic parameters such as fasting plasma glucose and glycosylated hemoglobin were analyzed using automated and standardized methods in our Department of Clinical Laboratory. The healthy controls without T2DM were frequency-matched to patients by age and gender. Exclusion criteria included those who had a history of cerebral stroke, heart stents, artificial pacemakers or other metal implants implanted in the body, or had malignant tumors, hepatopathy, end-stage renal disease, thyroid gland dysfunction, arthrophlogosis, carpal tunnel syndrome, or had taken special nutritional supplements such as protein powder in recent three months. Demographic characteristics (age, gender, income, education, and medical history) were collected by a general questionnaire. Height and weight were measured by standardized equipment wearing light indoor clothing without shoes. Body mass index (BMI) was calculated as body weight in kilograms divided by the square of body height in meters. The definition of overweight/obesity was based on criteria set by Working Group on Obesity in China^32^: obesity was defined as a BMI of ≥28 kg/m^2^ and overweight was defined as 24.0 ≤ BMI < 28 kg/m^2^. Visceral obesity was defined as the visceral fat area (VFA) ≥ 100 cm^2^ according to the Chinese Medical Association guidelines. Nephropathy was confirmed by serum creatinine >1.5 mg/dl in men and >1.2 mg/dl in women and peripheral neuropathy was identified by electromyography detection. Nutritional intake for all the adults was assessed with a modified food frequency questionnaire developed by Uenishi *et al*.^31^. The data collected from the questionnaire were uploaded to the clinical nutrition management software (Zhending Health Science Technology Co. Ltd, Shanghai, China) and the energy or protein intake level were calculated automatically and output on the report. Physical activity (PA) was assessed using a validated global PA questionnaire^33^ and measured in the form of metabolic equivalent (MET). The MET score was calculated according to the intensity and frequency of exercise performed per week for each participant. All questionnaire data were collected by trained interviewers. This study was approved by the Medical Ethics Committee of the Affiliated Hospital of Jiangsu University. All methods and analyses were carried out in accordance with the approved protocol and guidelines. Written informed consents were obtained from all study participants.

### Measurement of skeletal muscle mass, muscle strength, and physical performance

We recently developed a method for screening whole-body muscle mass using bioimpedance analysis (BIA). The BIA method provides simple, safe and reliable estimates of skeletal muscle mass in the elderly and has been validated for measuring appendicular skeletal muscle mass (ASM) in large cohorts^16^. For BIA, whole body composition was analyzed by a multi-frequency bioelectrical impedance analysis device (MC-180, TanitaCo., Ltd, Japan), which is an 8-point tactile electrode system. The participant was required to start the test after overnight fasting and remain seated for 5 minutes before the measurement. Each subject was trained to stand barefoot with their feet placed on the feet electrodes symmetrically in an upright position and their arms straight down, while keeping hands gripping on to the hand electrodes according to the instrument prompt. The segmental resistance was measured at six frequencies (1, 5, 50, 250, 500, and 1,000 kHz) and the device output included parameters composed of fat mass, fat free mass, ASM, trunk muscle mass, protein, mineral, total body water, intracellular water, extracellular water and visceral fat area. Finally, the skeletal muscle index (SMI) was calculated as ASM divided by the square of body height in meters.

Muscle strength was assessed by handgrip strength using a JAMAR Hydraulic Hand Dynamometer (Sammons Preston Rolyan, Chicago, IL, USA) in a standard posture as recommended by the American Society of Hand Therapist (ASHT)^34^. Handgrip strength measurement of each subject was done on both arms, repeated three times, and the maximum value was used. Physical performance was examined by 4-meter gait speed with each participant walking at daily speed twice and the time of completion recorded. The mean value was taken to calculate the gait speed.

### Diagnosis of sarcopenia and pre-sarcopenia

Sarcopenia was defined as presenting of both low muscle mass and function (strength or performance) following the AWGS criteria and pre-sarcopenia was defined as low muscle mass without impact on muscle strength or physical performance according to the AWGS criteria about the conceptual staging. Low muscle mass was defined as SMI < 7.0 kg/m^2^ in men and SMI < 5.7 kg/m^2^ in women. Low muscle strength was defined as handgrip strength <26 kg in men and <18 kg in women. Low physical performance was defined as gait speed <0.8 m/s.

### Statistical analysis

Continuous variables were expressed as mean ± standard deviation. Variables in normal distribution were compared using student’s t-test or one-way analysis of variance (ANOVA). If the data were not normally distributed, the Kruskal-Wallis test was used to compare the groups. Categorical variables were expressed as counts and percentages and were compared using chi-square test. The differences of prevalence of sarcopenia, pre-sarcopenia between T2DM patients and controls were compared using Cochran’s and Mantel-Haenszel test. Multivariate logistic regression model was used and Odds ratio and 95% confidence interval (CI) for sarcopenia was calculated to estimate the association of Type 2 diabetes and sarcopenia among older adults. All the tests were two-tailed and values of p < 0.05 were considered statistical significant. All the analyses were performed using SPSS 16.0 (SPSS Inc., Chicago, IL).

## Additional Information

**How to cite this article**: Wang, T. *et al*. Type 2 diabetes mellitus is associated with increased risks of sarcopenia and pre-sarcopenia in Chinese elderly. *Sci. Rep.* **6**, 38937; doi: 10.1038/srep38937 (2016).

**Publisher's note:** Springer Nature remains neutral with regard to jurisdictional claims in published maps and institutional affiliations.

## Figures and Tables

**Table 1:** Characteristics of T2DM patients and controls stratified by gender.

Parameters	Men			Women		
	T2DM Patients (n = 116)	Controls (n = 404)	*P*	T2DM Patients (n = 120)	Controls (n = 450)	*P*
Age (years)	68.4 ± 7.9	69.92 ± 8.12	0.385	68.86 ± 6.34	68.84 ± 6.33	0.985
Height (m)	1.67 ± 0.07	1.65 ± 0.07	0.024	1.57 ± 0.05	1.56 ± 0.06	0.593
Weight (kg)	67.86 ± 8.27	64.71 ± 13.98	0.196	59.53 ± 9.0	58.34 ± 7.57	0.43
BMI (kg/m^2^)	24.44 ± 2.52	23.76 ± 3.63	0.256	24.12 ± 3.43	23.8 ± 2.9	0.579
Trunk FM (kg)	9.26 ± 3.0	7.85 ± 3.8	**0.007**	11.55 ± 4.47	10.95 ± 3.62	0.41
AFM (kg)	6.27 ± 1.78	5.43 ± 2.22	**0.019**	8.21 ± 2.51	8.12 ± 2.13	0.83
Fat %	22.34 ± 5.1	19.78 ± 6.36	**0.017**	32.28 ± 6.91	32.05 ± 6.35	0.845
VFA (cm^2^)	139.6 ± 26.69	130.6 ± 38.4	0.158	84.2 ± 29.2^d^	81.1 ± 24.9	0.527
Overweight (%)	47.4	32.2	0.003	31.7	24.4	0.109
Obesity (%)	6	5.4	0.808	15.8	7.1	**0.003**
VSO (%)	93.1	48	<**0.001**	29.2	19.6	<**0.001**
Trunk MM (kg)	27.74 ± 2.78	27.06 ± 5.4	0.227	22.19 ± 1.79	21.49 ± 2.15	0.031
ASM (kg)	22.51 ± 3.21	21.73 ± 4.35	0.217	15.27 ± 1.54	15.51 ± 2.07	0.453
SMI (kg/m^2^)	8.03 ± 0.85	7.99 ± 1.15	0.824	6.18 ± 0.51	6.33 ± 0.8	0.215
HS (kg)	38.23 ± 7.88	38.7 ± 6.98	0.673	24.57 ± 4.37	25.25 ± 4.56	0.295
Gait speed (m/s)	1.08 ± 0.22	1.23 ± 0.18	<**0.001**	1.07 ± 0.26	1.26 ± 0.16	<**0.001**
FPG (mmol/l)	9.07 ± 3.34	5.36 ± 0.44	<**0.001**	9.44 ± 3.57	5.31 ± **0.48**	<**0.001**
HbA1C (%)	8.69 ± 1.91	5.93 ± 0.41	<**0.001**	8.88 ± 1.79	5.92 ± 0.38	<**0.001**
Diabetes duration (years)	9.31 ± 7.32	N/A	N/A	10.52 ± 7.43	N/A	N/A
Anti-diabetic medications (%)	85.7	N/A	N/A	88.1	N/A	N/A
Nephropathy (%)	21.2	6.8	<**0.001**	20.4	7.6	<**0.001**
Peripheral neuropathy (%)	78.3	9.4	<**0.001**	74.5	9.8	<**0.001**
Energy intake (kcal/d)	1610.87 ± 529.67	1785.33 ± 546.18	**0.036**	1483.51 ± 432.35	1576.38 ± 465.26	**0.041**
Protein intake (g/d)	54.36 ± 24.83	60.74 ± 26.18	0.085	46.89 ± 16.55	52.76 ± 20.15	0.261
Physical activity (MET_S_ min/week)	678(210~5624)	562(126~6013)	**0.031**	511(184~4137)	532(105~4304)	0.53

Notes: Data are expressed as the mean ± standard deviation or % and median. Abbreviations: Total FM = Total body fat mass; Trunk FM = Trunk fat mass; AFM = Appendicular fat mass; VFA = visceral fat area; VSO = visceral obesity; FFM = fat free mass; Total MM = total muscle mass; Trunk MM = Trunk muscle mass; ASM = appendicular skeletal muscle mass; SMI = skeletal muscle index; HS = handgrip strength; FPG = fasting plasma glucose; HbA1c = glycated hemoglobin; MET = metabolic equivalent.

**Table 2:** Prevalence of sarcopenia and pre-sarcopenia in T2DM patients and controls, overall and stratified by gender, n (%).

	T2DM patients				Controls				P value**
	Overall (n = 236)	Men (n = 116)	Women (n = 120)	*P* value*	Overall (n = 854)	Men (n = 404)	Women (n = 450)	*P* value*	
Sarcopenia	35 (14.8)	20 (17.2)	15 (12.5)	0.104	96 (11.2)	53 (13.1)	43(9.6)	0.1	0.035
Pre-sarcopenia	34 (14.4)	22 (19.0)	12 (10.0)	0.03	72 (8.4)	46 (11.4)	26(5.8)	0.002	0.002

Sarcopenia is defined as low skeletal muscle index (SMI) (less than 7.0 kg/m^2^ in men and 5.7 kg/m^2^ in women) and low muscle strength (handgrip strength <26 kg in men and <18 kg in women) or low physical performance(gait speed <0.8 m/s). Pre-sarcopenia is defined as low SMI but with normal muscle strength and normal physical performance. ^*^Analyzed by chi-square test, compared within gender; ^**^Comparing overall prevalence between patients and controls.

**Table 3:** Prevalence of sarcopenia in all study participants stratified by gender and age, n (%).

	60 ≤ age < 70		age ≥ 70		*P* value*
	Sarcopenia	Normal	Sarcopenia	Normal	
Men (n = 522)	22(8.3)	242(91.7)	51(19.8)	207(80.2)	＜0.001
Women (n = 568)	20(6.4)	294(93.6)	38(15.0)	216(85.0)	0.001
Total	42(7.3)	536(92.7)	89(17.4)	423(82.6)	＜0.001

^*^Analyzed by Cochran’s and Mantel-Haenszel test, comparisons of sarcopenia prevalence between age groups.

**Table 4:** Multivariable logistic regression analyses of T2DM and sarcopenia, pre-sarcopenia and low SMI among older adults in China, stratified by age.

	All participants (n = 1090)		OR (95% CI)*	60≤age < 70 (n = 578)		OR (95% CI)*	age ≥ 70 (n = 512)		OR (95% CI)*
	T2DM (n = 236)	Controls (n = 854)		T2DM (n = 152)	Controls (n = 426)		T2DM (n = 84)	Controls (n = 428)	
Sarcopenia	35 (14.8)	96 (11.2)	1.37 (1.02–2.03)	15 (9.9)	27 (6.3)	1.47 (0.61–2.75)	20 (23.8)	69 (16.1)	1.51 (0.72–2.25)
Pre-sarcopenia	34 (14.4)	72 (8.4)	1.73 (1.10–2.83)	16 (10.5)	28 (6.6)	1.57 (0.74–2.79)	18 (21.4)	44 (10.3)	2.17 (1.11–4.03)
Low SMI	69 (29.2)	168 (19.6)	1.64 (1.25–2.07)	31 (21.1)	55 (12.9)	1.41 (0.87–2.35)	38 (45.2)	113(26.4)	2.23 (1.18–2.85)

^*^Adjusted by age, gender, anti-diabetic medication, energy intake, protein intake, physical activity, and visceral fat area. Categorical variables are presented as numbers and percentages. SMI: skeletal muscle index.
